# The interplay of long non-coding RNAs and MYC in cancer

**DOI:** 10.3934/biophy.2015.4.794

**Published:** 2015-12-01

**Authors:** Michael J. Hamilton, Matthew D. Young, Silvia Sauer, Ernest Martinez

**Affiliations:** Department of Biochemistry, University of California, Riverside, CA 92521, USA

**Keywords:** lncRNA, MYC, epigenetics, chromatin, transcription, cancer

## Abstract

Long non-coding RNAs (lncRNAs) are a class of RNA molecules that are changing how researchers view eukaryotic gene regulation. Once considered to be non-functional products of low-level aberrant transcription from non-coding regions of the genome, lncRNAs are now viewed as important epigenetic regulators and several lncRNAs have now been demonstrated to be critical players in the development and/or maintenance of cancer. Similarly, the emerging variety of interactions between lncRNAs and MYC, a well-known oncogenic transcription factor linked to most types of cancer, have caught the attention of many biomedical researchers. Investigations exploring the dynamic interactions between lncRNAs and MYC, referred to as the lncRNA-MYC network, have proven to be especially complex. Genome-wide studies have shown that MYC transcriptionally regulates many lncRNA genes. Conversely, recent reports identified lncRNAs that regulate MYC expression both at the transcriptional and post-transcriptional levels. These findings are of particular interest because they suggest roles of lncRNAs as regulators of MYC oncogenic functions and the possibility that targeting lncRNAs could represent a novel avenue to cancer treatment. Here, we briefly review the current understanding of how lncRNAs regulate chromatin structure and gene transcription, and then focus on the new developments in the emerging field exploring the lncRNA-MYC network in cancer.

## 1. Introduction

In recent years, the investigations exploring the importance of how long non-coding RNAs (lncRNAs) influence epigenetic modifications and chromatin structure has truly been paradigm shifting in our fundamental understanding of how transcription is regulated in higher eukaryotes. Once considered to be “transcriptional noise” inherent to the large genomes of higher eukaryotic organisms, lncRNAs are now viewed as critical regulators of complex genomes and have added another layer of complexity to the molecular mechanisms that govern gene regulation. In humans there is ~ 2 times more genes that produce lncRNAs, an estimated ~ 48,000 lncRNA genes [1], than protein-coding genes, and only a very small fraction of these lncRNA genes have been characterized [1].

While lncRNAs are only a subset of the non-coding transcriptome, over the last several years these mysterious RNAs have stepped into the limelight. In particular, a topic of great interest has been how dysregulation of lncRNAs leads to the inappropriate epigenetic regulation of critical genes that are involved in the development and/or maintenance of cancer. Recent evidence suggests that MYC, a well-studied oncogenic transcription factor that is deregulated in most types of cancer and controls many cellular processes, including cell growth, metabolism, proliferation, differentiation and apoptosis [2–6], is an important mediator in the transcription of lncRNAs [7,8]. In turn, new evidence suggests that lncRNAs can also control the expression of MYC [9]. In this review, we briefly discuss our current understanding of the basic features of lncRNAs and how they regulate the epigenetic landscape and then focus on the emerging dynamic relationships between MYC and several lncRNAs as they pertain to cancer.

## 2. Characteristics of lncRNAs: Structure and Function

The generally accepted definition of a lncRNA is an RNA molecule longer than 200 nucleotides that does not code for a protein [10]. With the arrival of genome-wide platforms, such as microarrays and next-generation sequencing, and more sophisticated computational analyses of genome-wide data, the exploration of the non-coding transcriptome has developed a strong foothold in molecular research laboratories [11–14]. In a recent publication, it was estimated that there are ~ 110,000 different lncRNA transcripts within the human genome, with ~ 80,000 of these considered to be high confidence lncRNA transcripts, representing ~ 48,000 genes [1]. Through the use of sophisticated computational analyses, these high confidence lncRNAs transcripts were shown to have very limited or no appreciable coding potential [1]. LncRNAs share many similarities to protein-coding transcripts. LncRNAs undergo similar co-transcriptional and post-transcriptional processing. Many lncRNA transcripts are transcribed by RNA polymerase II (although some are transcribed by RNA polymerase III) [15], they share the same canonical splice sites and polyadenylation terminal signals and frequently contain a 5′ cap and a polyadenylated 3′-end [16,17].

**Figure 1:**
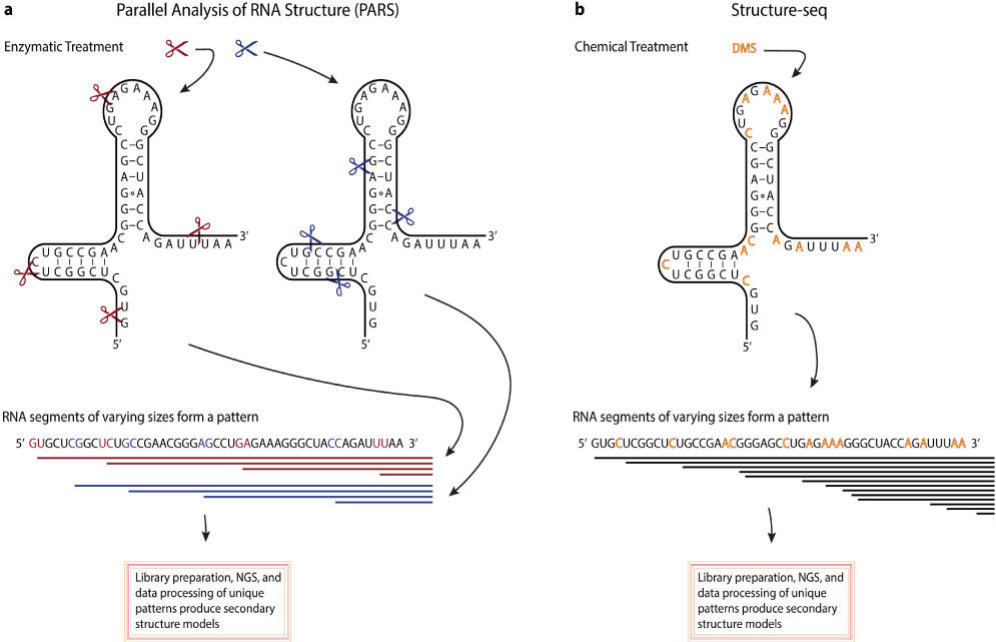
Overview of two methodologies used to determine RNA secondary structure. A. Parallel analysis of RNA structure (PARS) uses an *in vitro* enzymatic treatment with single strand (S1 nuclease, red scissor) and double strand (RNase V1, blue scissor) cutters to generate two pools of digested RNA. Once digested, adaptor sequences are ligated to the cleavage sites, converted into a cDNA library and subject to next-generation sequencing (NGS). Cleavages sites, identified from the sequencing data, will provide the locations of double stranded RNA regions (seen from the RNase V1 cleavage sites) or single stranded regions (seen from the S1 nuclease cleavage sites). Collectively, from these data secondary structure of RNA molecules can be determined. B. An *in vivo* chemical treatment, named Structure-seq, uses DMS to selectively methylate available adenines and cytosines (denoted by red letters). Reverse transcriptase activity stops one nucleotide before reaching the methylated adenine or cytosine. A cDNA library is constructed and subject to NGS. As a result, the signature of discernable stop sites can be used to infer secondary structure from NGS data.

Importantly, there are also some notable dissimilarities between lncRNAs and mRNAs. LncRNAs can undergo some unconventional processing [18,19,20] and tend to have a higher degree of tissue-specific expression relative to protein-coding genes [14,21,22]. Additionally, the primary sequences of lncRNAs tend to be less conserved across species [23]. These data suggest that the structure (rather than sequence) of lncRNAs may be of greater importance when exploring the function of lncRNAs, but this topic of RNA biology remains challenging. LncRNAs that have been extensively studied, such as HOTAIR and MALAT1, have provided some of the initial insights into the importance of the structural features of lncRNAs [23–27]. Recent evidence has shown that secondary structural elements of lncRNAs that are evolutionarily conserved contain important protein-binding domains [27]. Several methodologies have been developed in an effort to determine the secondary and tertiary structures of RNA molecules [28]. Some of the most noteworthy techniques have used either specific nucleolytic enzymes or chemical modifications of the RNA molecules followed by sequencing [29–33]. Once sequenced, the secondary structure of the RNA molecules can be determined from the ends of the reads using advanced computational tools (Figure 1). For example, the technique frequently referred to as Structure-seq employs the use of dimethyl sulfate (DMS) which penetrates the cells and methylates N1 of adenines and N3 of cytosines when not involved in Watson-Crick base pairing [29,35]. Reverse transcription of the DMS-methylated RNA, results in reverse transcriptase stopping at the methylated bases generating cDNAs of different lengths that are then sequenced. From these data, the secondary structure of RNA molecules can be determined on a genome-wide level using computational models. However, most techniques for the structural analysis of RNA molecules involve *in vitro* conditions that may not retain the structural characteristics of lncRNAs *in vivo* [36]. In spite of the challenges in RNA structural biology, the elucidation of lncRNA structure appears to be of vital importance, if we are to fully understand the functional capabilities of lncRNAs.

**Figure 2:**
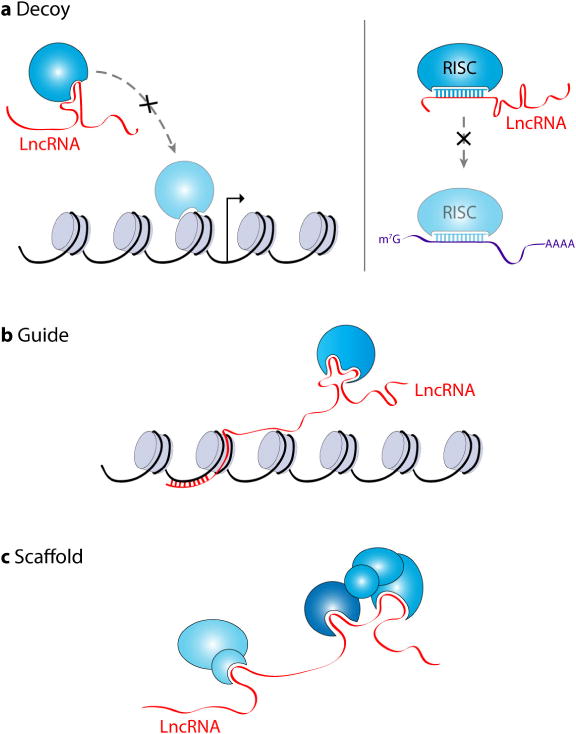
Functional categories of lncRNAs. There are three broad functional classifications for lncRNAs. A. LncRNAs can act as decoys sequestering either proteins and/or regulatory RNAs, such as miRNAs, away from their targets or cellular locations. B. LncRNAs can also be key players in the recruitment of proteins, such chromatin-modifying enzymes, to specific genomic locations thereby influencing transcriptional events. C. LncRNAs can provide a platform or scaffold to facilitate different molecular interactions, such as protein-protein interactions.

LncRNAs have a myriad of functions within eukaryotic cells. One of the best-understood and studied functions of lncRNAs is how they modulate gene expression. LncRNAs have been described as “fine-tuners” of gene regulatory networks regulating gene expression both at the transcriptional and post-transcriptional levels via a variety of distinct mechanisms [37,38]. LncRNAs can be cisacting, regulating chromatin structure and transcription of neighboring genes, and/or trans-acting, regulating the transcription of genes at distant locations within the genome [10,38]. There are three broad functional classifications for lncRNAs; they can serve as decoys, scaffolds and/or guides (Figure 2). In simple terms, lncRNAs that serve as decoys can associate with both regulatory RNAs and proteins, such as miRNAs, DNA-binding proteins and histone-modifying enzymes, and prevent their binding to specific target mRNAs or chromatin loci and/or inhibit their enzymatic activity [10,37,39,40]. In a recent example, MALAT1 was shown to mediate the mRNA levels of serum release factor (SRF), an important transcription factor in myogenesis, by acting as a sponge or competing endogenous RNA (ceRNA) for miR-133 [40]. Scaffold lncRNAs provide a platform onto which different molecular interactions can occur, such as protein-protein interactions, including interactions of distant chromatin loci [41]. For example, HOTAIR has been demonstrated to aid in protein ubiquitination by acting as a scaffold for E3 ubiquitin ligases containing Dzip3 and Mex3b RNA-binding domains and also their substrates, Ataxin-1 and Snurportin-1, respectively [41]. Lastly, guide lncRNAs aid in the recruitment of protein complexes to specific locations within the cell, such as recruitment of epigenetic modifying enzymes to specific genes [10,37,38,42]. The Xist lncRNA and its role in dosage compensation is a prime example of a guide lncRNA. Xist has been shown to interact with the SHARP-SMRT complex and recruit it to the X chromosome, thereby activating HDAC3 leading to histone deacetylation and exclusion of RNA polymerase from the X chromosome [42]. As more lncRNAs are characterized, we suspect these functional classifications will change with the discovery of novel lncRNA cellular functions. All three of these broad functional classifications of lncRNAs are found within the lncRNA-MYC network, described below.

## 3. The lncRNA-MYC Network

Over the last decade as lncRNAs have been drawing the attention of more researchers, as too has the lncRNA-MYC network gained the attention of many investigators. In two recent reviews, the lncRNA-MYC network has been described: either by examining how lncRNAs influence MYC expression [9], or selectivity summarizing some of the interactions seen within the human lncRNA-MYC regulatory network [43]. Here, we will further expand on what is known about the lncRNA-MYC network by providing a comprehensive summary of the molecular interactions within this regulatory network (Table 1), with an emphasis on recent developments in the field demonstrating functional relationships between cancer-associated lncRNAs and MYC.

### 3.1. PVT1

**Figure 3:**
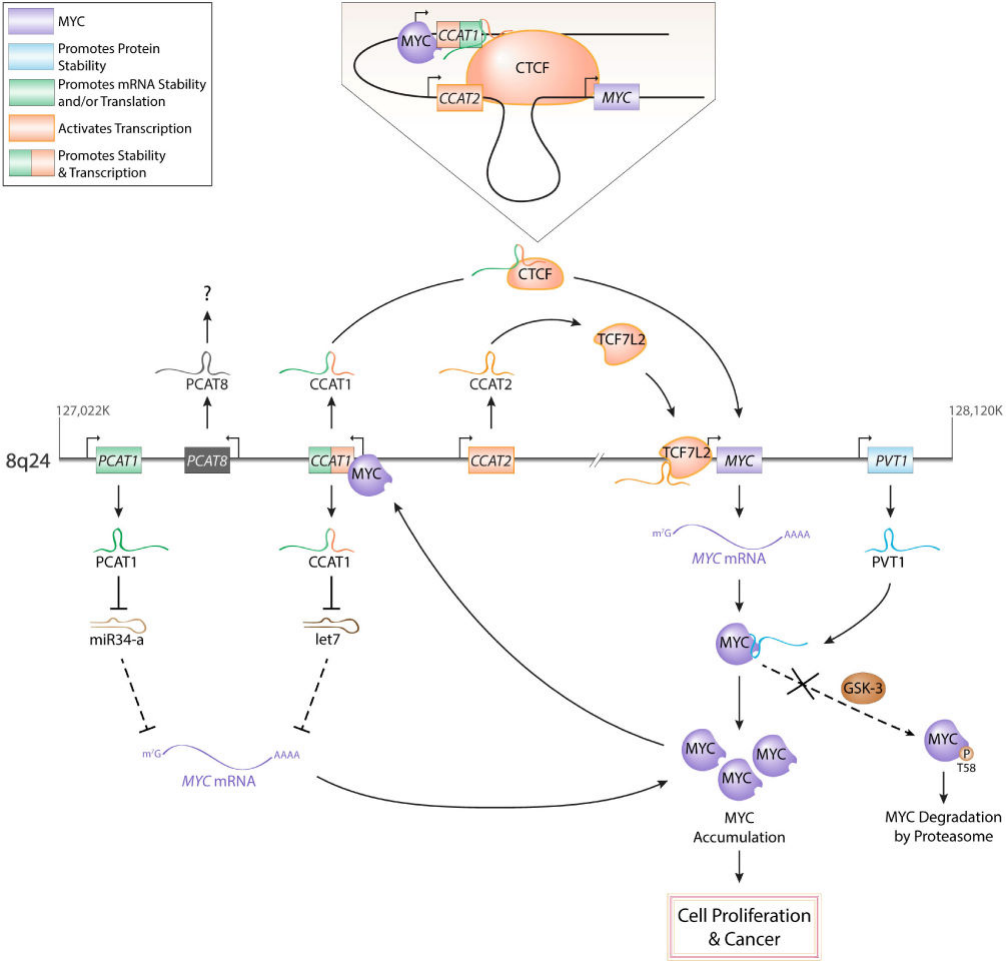
The cancer-specific molecular interactions of the lncRNA-MYC network at the 8q24 genomic region. CCAT1 both transcriptionally (through chromatin interactions) and post-transcriptionally (through titration of let-7) regulates *MYC* expression. CCAT2 stimulates TCFL2-mediated transcription of the *MYC* locus. PCAT1 prevents miR34-a mediated translational repression. PVT1 binds to MYC preventing threonine-58 phosphorylation by glycogen synthase kinase 3 (GSK3) and subsequent MYC degradation. Collectively, all of the flanking lncRNAs promote the accumulation of MYC; therefore, when these lncRNA are inappropriately upregulated, MYC-dependent malignancies can develop.

We will begin by describing recent findings suggesting a reciprocal relationship between MYC (formerly c-MYC) and a well-known lncRNA, known as PVT1. Both the *MYC* and *PVT1* genes are located in the 8q24 chromosomal region, which is frequently referred to as a “gene desert” because it contains few protein-coding genes (Figure 3). However, several lncRNA genes have been discovered within this region. The 8q24 region has also been of particular interest because it is a frequent region of genomic alterations, including amplifications and translocation breakpoints, in several different types of cancer [44]. Moreover, aberrant overexpression of PVT1 has been discovered in many different human cancers [45]. As previous mentioned, MYC is an oncogenic transcription factor and can either activate or repress transcription [46]. In a recent study, *PVT1* was shown to contain two non-canonical MYC-binding sites, which were found to be important for the binding of both MYC and its paralog MYCN (formerly N-MYC) to the promoter region of *PVT1*, with changes in H4 acetylation and *PVT1* mRNA production correlating with changes in MYCN occupancy at the PVT1 promoter [47]. As suggested by the authors, this study demonstrates that *PVT1* is a likely downstream target of MYCN. Conversely, PVT1 lncRNA has been shown to be important in the regulation of MYC expression. Through the use of chromosome engineering in mice and both loss and gain-of-function analyses in different human cancer cell lines, it was demonstrated that PVT1 was required for high MYC protein expression, via its capacity to protect MYC from phosphorylation and subsequent degradation [48]. PVT1 is an exceptionally interesting lncRNA, both in its ability to physically interact with and regulate MYC and its pivotal roles in many cancers, making it an attractive therapeutic target to combat different cancers. For a more extensive review of PVT1 and its oncogenic features, we will refer to a recent review by Colombo et al. [45].

### 3.2. The CCAT family

The colon cancer associated transcripts (CCATs) are a collection of lncRNAs located on different chromosomes that have been both associated with and functionally demonstrated to be involved in the development of human colorectal cancers (CRC). Specifically, three of the best-characterized CCAT lncRNAs are CCAT1 (also known as CARLo5), CCAT2 and CCAT6. CCAT6, also known as MYCLo2, will be discussed below in the MYCLos section. While the *CCAT6* gene is located on chromosome 7, *CCAT1* and *CCAT2* are located in the gene desert region of 8q24, near *MYC* and *PVT1*. With the use of genome-wide association (GWA) studies, the 8q24 region has been implicated in CRC [49,50,51]. From these GWA studies, CCAT1 was later identified and characterized as being a highly specific marker for CRC [52].

The interplay between MYC and CCAT1 involves many complex molecular interactions. Contained within the 8q24 region are several chromatin-looping interactions that have been shown to be tissue-specific [53] and have been suggested to regulate MYC expression [53–58]. One of the most studied structural elements found in the 8q24 region is an enhancer region located ~ 335 kb upstream of *MYC*, frequently referred to as MYC-335 [55,56,57]. Located ~180 kb upstream of MYC-335 is *CCAT1*, and this region is considered to be a super-enhancer (Figure 3). A recent study showed a long-range physical interaction between MYC-335 and the promoter of *CCAT1*, suggesting that MYC-335 is important for CCAT1 expression [54]. Moreover, it was later demonstrated that a long isoform of CCAT1, referred to as CCAT1-L, was important in the maintenance of the chromatin interaction via its role in the recruitment of a transcription factor, called CCCTC-Binding factor or CTCF [58]. Moreover, CCAT1 has been suggested to also regulate MYC post-transcriptionally. Deng et al., found that CCAT1 was deregulated in hepatocellular carcinoma, and CCAT1 expression correlated with the progression of the malignancy and poor prognosis [59]. With the use of RNA immunoprecipitation, CCAT1 was discovered to function as a let-7 miRNA sponge, thereby disinhibiting MYC [59]. Adding to the complexity, MYC has been shown to bind to the promoter of *CCAT1* and upregulate its expression and promote proliferation and invasion of colon and gastric cancer cells [60,61].

Also important to the regulation of MYC expression is the CCAT2 lncRNA. CCAT2 is transcribed from MYC-335, described above, and CCAT2 is overexpressed in CRC and has been shown to promote tumor growth and metastasis [62]. Moreover, in the same study, CCAT2 was also shown to upregulate transcription of *MYC* through a physical interaction with TCF7L2 [62]. However, further investigation is needed to determine mechanistically how CCAT2 is stimulating TCF7L2-mediated transcription of MYC. Recently, additional CCAT lncRNAs have been discovered; however, it remains unclear whether these novel CCAT lncRNAs are part of the lncRNA-MYC regulatory network [63]. Altogether, the CCAT lncRNA family is proving to be complex and important in the involvement of colorectal cancer, and possibly other cancers, and in the regulation of MYC expression.

### 3.3. MYCLos

MYCLos is a collective term for several lncRNAs included within the CCAT family, coined by a research group examining the importance of these lncRNAs in human CRC. In the original study, conducted by Kim et al., a microarray analysis was used to profile ~33,000 lncRNAs in both normal and CRC samples [63]. Their results revealed thousands of lncRNAs to be differentially expressed, including the CCAT1 and CCAT2 lncRNAs that had previously been demonstrated to be important in several stages of CRC [52,54,58,62,64,65]. To further narrow their search and to isolate the lncRNAs that were both differentially expressed in CRC and regulated by MYC, they examined the effects of MYC knockdown in different CRC cell lines. From these experiments, they identified three lncRNAs, referred to as MYCLo1, MYCLo2 (also known as CCAT6), and MYCLo3, that were transcriptionally upregulated by MYC. They later confirmed that MYCLos had influential roles in cell proliferation and cell cycle progression by regulating the expression of *CDKN1A* and *CDKN2B*, known gene targets of MYC. In a follow-up study by the same research group, three additional lncRNAs were identified, named MYCLo4, MYCLo5, and MYCLo6, and were repressed by MYC. Similar to MYCLos1–3, MYCLos4–6 were also found to influence cell proliferation and cell cycle progression, by regulating the expression of MYC target genes [66]. Collectively, MYCLos are a newly identified class of MYC-regulated lncRNAs, with some of them having an oncogenic role (MYCLos 1–3) and others having a tumor suppressor role (MYCLos 4–6). In the future, it will be important to determine how universal these lncRNAs are to the functions of MYC and whether a similar regulation of MYCLos by MYC is observed in other cancers.

### 3.4. The PCAT family

The prostate cancer associated transcripts (PCATs) are another class of lncRNAs within the lncRNA-MYC network. Three of the better-characterized PCAT lncRNAs are PCAT1, PCAT8 (also known as PRNCR1 and CARLo-3) and PCAT9 (also known as PCGEM1). While *PCAT9* is located on chromosome 2, *PCAT1* and *PCAT8* are located ~ 715 kb and ~ 645 kb upstream of *MYC*, respectively. As mentioned above, lncRNAs have been shown to regulate the transcription of the *MYC* gene and the stability of the MYC protein. More recent evidence suggests that lncRNAs may also influence MYC protein expression at the mRNA level. In a recent study in prostate cancer cells, it was shown that PCAT1 attenuates the downregulation of MYC protein expression (but not mRNA amount or stability) by interfering with miR-34a [67], a known miRNA that regulates MYC expression by targeting the MYC mRNA 3′UTR [68,69,70]. Although many lncRNAs act as sponges to sequester miRNAs away from their mRNA targets [71,72], the investigators were unable to identify any putative miR-34a binding site in PCAT1. Therefore, it was suggested that PCAT1 indirectly affects the miR-34a post-transcriptional regulation of MYC [67]. While PCAT1 does appear to be directly involved in the lncRNA-MYC network, it is unclear if PCAT8 is also part of this network; however, PCAT8 has been associated with both prostate and colorectal cancers [73,74].

In a study by Hung et al., PCAT9, also known as prostate cancer gene expression marker 1 (PCGEM1), was found to be an important transcriptional mediator of many metabolic pathways in prostate cancer cells [75]. With chromatin isolation by RNA purification (ChIRP), a technique developed to examine specific RNA-DNA interactions [76], it was demonstrated that PCAT9 physically interacts with the promoters of metabolic genes, and that PCAT9 expression affected cell-cycle progression and proliferation [75]. Also discovered, PCAT9 was found to bind to MYC and that upon knockdown of PCAT9 recruitment of MYC to metabolic genes was diminished [75]. To our knowledge, PCAT9 is the only lncRNA that has been shown to bind to MYC and promote its transactivation activity thereby affecting the metabolism of cancer cells.

### 3.5. GAS5

The growth arrest-specific 5 (GAS5) lncRNA is a functionally diverse lncRNA [77], that is transcribed from chromosome 1. GAS5 has been suggested to be a tumor suppressor, implicated in several human cancers [78–82]. While PCAT1 disinhibited the translation of MYC mRNA, GAS5 has been demonstrated to interfere with the translation of MYC mRNA. In a recent study, GAS5 was shown to bind to both the eIF4E translation initiation factor and the MYC mRNA thereby inhibiting translation of MYC [82]. However, further investigation is needed to determine mechanistically how GAS5 is suppressing the translation of MYC mRNA. This study provides another example of the diversity of mechanisms by which lncRNAs regulate the expression of MYC.

### 3.6. GHET1

Gastric carcinoma proliferation enhancing transcript 1 (GHET1) is an unspliced lncRNA transcribed from chromosome 7 that has been implicated in gastric and bladder cancers [83,84]. First discovered by Yang et al., GHET1 was found to be upregulated in gastric carcinoma clinical samples and higher levels of GHET1 expression correlated with a poor survival rate [84]. Knockdown of GHET1 was shown to inhibit proliferation rates of gastric carcinoma cells. Conversely, overexpression of GHET1 promoted cell proliferation rates *in vitro* and tumor growth *in vivo*. With the use of different immunoprecipitation techniques, GHET1 was shown to physically interact with insulin-like growth factor 2 mRNA binding protein 1 (IGF2BP1) and also promote the binding of IGF2BP1 to MYC mRNA aiding in its stabilization [84]. The MYC mRNA is a very unstable mRNA that is rapidly degraded, and IGF2BP1 is part of a protein complex that has been shown to promote its stability [85,86]. In the future, it would be interesting to see if GHET1 maintains this same mechanistic relationship with MYC in other malignancies.

### 3.7. H19

Imprinted maternally expressed transcript, known as H19, is a lncRNA expressed only from the maternal allele on chromosome 11 that has been shown to be essential for human tumor growth and metastasis [87,88]. Moreover, H19 has been demonstrated to be functionally important in several human cancers [89–94]. While it has been known for many years that H19 is a key player in many human malignancies, it was only recently that a functional link between H19 and MYC had been discovered. MYC was found to bind to E-boxes located in the *H19* promoter and assist in histone acetylation, thereby promoting H19 expression [94]. Many of these findings were recapitulated in a later study [93]. Interestingly, H19 is predominantly a cytoplasmic lncRNA, and recently has been demonstrated to be important in muscle differentiation by acting as a molecular sponge for the let-7 miRNA [96]. Furthermore, the role of H19 in metastasis was elucidated later in ovarian cancer cells were H19 was discovered to interfere with let-7 mediated downregulation of MYC mRNA and protein levels [97]. Collectively, H19 is one of the most pervasive dysregulated lncRNAs seen in human cancer, and to date it is one of only a few lncRNAs that feeds into a positive feedback loop with MYC, by being transcriptionally upregulated by MYC and post-transcriptionally disinhibiting MYC mRNA degradation.

### 3.8. TUSC8

A relatively uncharacterized lncRNA, referred to as tumor suppressor candidate 8 (TUSC8) located on chromosome 13 has also be suggested to modulate the expression of MYC. In a study by Liao et al., TUSC8 was found to be downregulated in cervical cancer, and TUSC8 expression was found to correlate with the progression of the cervical cancer and patient survival rate. In HeLa, SiHA and HCC94 cells, overexpression of TUSC8 was discovered to diminish both MYC mRNA and protein levels and decrease proliferation rates, while knockdown of TUSC8 had an opposite effect in both MYC expression and proliferation rates [98]. However, the mechanisms of how TUSC8 regulates the expression of MYC is unclear and these observed effects on MYC expression could potentially be indirect.

## 4. Conclusion

Our understanding of the dynamic regulatory relationship between lncRNAs and MYC remains in its infancy. However, just within the past year there have been several studies exploring this potentially invaluable relationship found within many human cancers. It is not surprising that MYC would transcriptionally regulate many lncRNAs, and it is especially interesting that MYC oncogenic functions could be mediated through the regulation of specific lncRNAs. Given the crucial role of MYC in many cancers, these findings suggest that MYC-regulated lncRNAs and also lncRNAs that regulate MYC could be potential valuable targets in the treatment of many human cancers. MYCN is another interesting protein of the lncRNA-MYC network that is garnering attention. New studies have been conducted exploring a functional connection between MYCN and lncRNAs implicated in cancer [99–103]. Given its importance in the nervous system and mesenchymal tissues [104,105], like MYC, MYCN could also mediate some of its oncogenic functions through the regulation of lncRNAs. Currently, we are still left with many unanswered questions concerning the importance of the lncRNA-MYC regulatory network in the development and/or maintenance of cancer. Specifically, it would be interesting to know how pervasive these regulatory networks are and whether the same or distinct molecular interactions exist in different malignancies. Given the sheer number of different lncRNA genes/loci, which give rise to an even larger number of lncRNA transcripts, and the fact that many of these lncRNAs are expressed both in a temporal and tissue-specific manner [14,21,22], one could postulate the existence of many more lncRNAs that could be regulated by MYC in a context-dependent manner. Altogether, future investigations in understanding this complex regulatory network could serve to provide critical insights in the biology underlying the many different types of cancers.

## Figures and Tables

**Table 1:** Summary of the participants of the lncRNA-MYC network.

LncRNA	Aliases	Functional category	Chromosome	Regulated by MYC	Regulates MYC	Classification	Function	References
PVT1	LINC0007, onco-lncRNA-100	Decoy	8	Upregulated	Yes	Oncogene	Binds MYC preventing phosphorylation and degradation	[45,47,48]

**CCAT Family**								

CCAT1	CARLo5, onco-lncRNA-40	Guide, scaffold, decoy	8	Upregulated	Yes	Oncogene	Facilitates transcription of *MYC*, titrates let-7 away preventing *MYC* degradation	[52,54,58–61,63–65]
CCAT2	NCCP1, LINC00873	Guide	8	Unknown	Yes	Oncogene	Facilitates transcription of *MYC* via the stimulation of TCF7L2	[62]
MYCLo1	AK021907	Unknown	20	Upregulated	Unknown	Oncogene	–	[63]
MYCLo2	CCAT6, AC074389.9	Unknown	7	Upregulated	Unknown	Oncogene	–	[63]
MYCLo3	KTN1-AS1, C14orf33	Unknown	14	Upregulated	Unknown	Oncogene	–	[63]
MYCLo4	JX046912	Unknown	9	Downregulated	Unknown	Tumor suppressor	–	[66]
MYCLo5	JX046913, JX046914	Unknown	3	Downregulated	Unknown	Tumor suppressor	–	[66]
MYCLo6	JX046915	Unknown	3	Downregulated	Unknown	Tumor suppressor	–	[66]

**PCAT Family**								

PCAT1	PCA1	Unknown	8	Unknown	Yes*	Oncogene	Interferes with miR34-a preventing MYC translational repression	[67]
PCAT9	PCGEM1, LINC00071	Unknown	2	Unknown	Yes	Oncogene	Increases MYC transactivation activity	[75]

GAS5	SNHG2	Unknown	1	Unknown	Yes	Tumor suppressor	Prevents translation of *MYC* mRNA	[82]
GHET1	–	Guide, scaffold	7	Unknown	Yes	Oncogene	Promotes stability of *MYC* mRNA via the recruitment of IGF2BP1	[84]
H19	ASM, BWS, WT2, ASM1, D11S813E, LINC00008	Decoy	11	Upregulated	Yes	Tumor suppressor/oncogene	Titrates let-7 away preventing MYC mRNA degradation	[93,94,97]
TUSC8	XLOC_010588, LINC01071	Unknown	13	Unknown	Yes*	Tumor suppressor	–	[98]

* Unclear whether effect is direct or indirect

## References

[1] Volders PJ, Verheggen K, Menschaert G (2015). An update on LNCipedia: a database for annotated human lncRNA sequences. Nucleic Acids Res.

[2] Amati B, Frank SR, Donjerkovic D (2001). Function of the c-Myc oncoprotein in chromatin remodeling and transcription. Biochim Biophys Acta.

[3] Bretones G, Delgado MD, Leon J (2015). Myc and cell cycle control. Biochim Biophys Acta.

[4] Dang CV (2013). MYC, metabolism, cell growth, and tumorigenesis. Cold Spring Harb Perspect Med.

[5] McMahon SB (2014). MYC and the control of apoptosis. Cold Spring Harb Perspect Med.

[6] Vennstrom B, Sheiness D, Zabielski J (1982). Isolation and characterization of c-myc, a cellular homolog of the oncogene (v-myc) of avian myelocytomatosis virus strain 29. J Virol.

[7] Zheng GX, Do BT, Webster DE (2014). Dicer-microRNA-Myc circuit promotes transcription of hundreds of long noncoding RNAs. Nat Struct Mol Biol.

[8] Winkle M, van den Berg A, Tayari M (2015). Long noncoding RNAs as a novel component of the Myc transcriptional network. FASEB J.

[9] Xiang JF, Yang L, Chen LL (2015). The long noncoding RNA regulation at the MYC locus. Curr Opin Genet Dev.

[10] Rinn JL, Chang HY (2012). Genome regulation by long noncoding RNAs. Annu Rev Biochem.

[11] Kapranov P, Cawley SE, Drenkow J (2002). Large-scale transcriptional activity in chromosomes 21 and 22. Science.

[12] Rinn JL, Euskirchen G, Bertone P (2003). The transcriptional activity of human Chromosome 22. Genes Dev.

[13] Guttman M, Garber M, Levin JZ (2010). Ab initio reconstruction of cell type-specific transcriptomes in mouse reveals the conserved multi-exonic structure of lincRNAs. Nat Biotechnol.

[14] Cabili MN, Trapnell C, Goff L (2011). Integrative annotation of human large intergenic noncoding RNAs reveals global properties and specific subclasses. Genes Dev.

[15] Wu J, Okada T, Fukushima T (2012). A novel hypoxic stress-responsive long non-coding RNA transcribed by RNA polymerase III in Arabidopsis. RNA Biol.

[16] Derrien T, Johnson R, Bussotti G (2012). The GENCODE v7 catalog of human long noncoding RNAs: analysis of their gene structure, evolution, and expression. Genome Res.

[17] Gardini A, Shiekhattar R (2015). The many faces of long noncoding RNAs. FEBS J.

[18] Wilusz JE, Spector DL (2010). An unexpected ending: noncanonical 3′ end processing mechanisms. RNA.

[19] Zhang Y, Yang L, Chen LL (2014). Life without A tail: new formats of long noncoding RNAs. Int J Biochem Cell Biol.

[20] Peart N, Sataluri A, Baillat D (2013). Non-mRNA 3′ end formation: how the other half lives. Wiley Interdiscip Rev RNA.

[21] Ravasi T, Suzuki H, Pang KC (2006). Experimental validation of the regulated expression of large numbers of non-coding RNAs from the mouse genome. Genome Res.

[22] Djebali S, Davis CA, Merkel A (2012). Landscape of transcription in human cells. Nature.

[23] He S, Liu S, Zhu H (2011). The sequence, structure and evolutionary features of HOTAIR in mammals. BMC Evol Biol.

[24] Brown JA, Bulkley D, Wang J (2014). Structural insights into the stabilization of MALAT1 noncoding RNA by a bipartite triple helix. Nat Struct Mol Biol.

[25] Brown JA, Valenstein ML, Yario TA (2012). Formation of triple-helical structures by the 3′-end sequences of MALAT1 and MENbeta noncoding RNAs. Proc Natl Acad Sci U S A.

[26] Smith MA, Gesell T, Stadler PF (2013). Widespread purifying selection on RNA structure in mammals. Nucleic Acids Res.

[27] Somarowthu S, Legiewicz M, Chillon I (2015). HOTAIR forms an intricate and modular secondary structure. Mol Cell.

[28] Mortimer SA, Kidwell MA, Doudna JA (2014). Insights into RNA structure and function from genome-wide studies. Nat Rev Genet.

[29] Ding Y, Tang Y, Kwok CK (2014). In vivo genome-wide profiling of RNA secondary structure reveals novel regulatory features. Nature.

[30] Kertesz M, Wan Y, Mazor E (2010). Genome-wide measurement of RNA secondary structure in yeast. Nature.

[31] Lucks JB, Mortimer SA, Trapnell C (2011). Multiplexed RNA structure characterization with selective 2′-hydroxyl acylation analyzed by primer extension sequencing (SHAPE-Seq). Proc Natl Acad Sci U S A.

[32] Seetin MG, Kladwang W, Bida JP (2014). Massively parallel RNA chemical mapping with a reduced bias MAP-seq protocol. Methods Mol Biol.

[33] Underwood JG, Uzilov AV, Katzman S (2010). FragSeq: transcriptome-wide RNA structure probing using high-throughput sequencing. Nat Methods.

[34] Aviran S, Trapnell C, Lucks JB (2011). Modeling and automation of sequencing-based characterization of RNA structure. Proc Natl Acad Sci U S A.

[35] Rouskin S, Zubradt M, Washietl S (2014). Genome-wide probing of RNA structure reveals active unfolding of mRNA structures in vivo. Nature.

[36] Novikova IV, Hennelly SP, Sanbonmatsu KY (2013). Tackling structures of long noncoding RNAs. Int J Mol Sci.

[37] Fatica A, Bozzoni I (2014). Long non-coding RNAs: new players in cell differentiation and development. Nat Rev Genet.

[38] Batista PJ, Chang HY (2013). Long noncoding RNAs: cellular address codes in development and disease. Cell.

[39] Batista PJ, Chang HY (2013). Cytotopic localization by long noncoding RNAs. Curr Opin Cell Biol.

[40] Han X, Yang F, Cao H (2015). Malat1 regulates serum response factor through miR-133 as a competing endogenous RNA in myogenesis. FASEB J.

[41] Yoon JH, Abdelmohsen K, Kim J (2013). Scaffold function of long non-coding RNA HOTAIR in protein ubiquitination. Nat Commun.

[42] McHugh CA, Chen CK, Chow A (2015). The Xist lncRNA interacts directly with SHARP to silence transcription through HDAC3. Nature.

[43] Deng K, Guo X, Wang H (2014). The lncRNA-MYC regulatory network in cancer. Tumour Biol.

[44] Beroukhim R, Mermel CH, Porter D (2010). The landscape of somatic copy-number alteration across human cancers. Nature.

[45] Colombo T, Farina L, Macino G (2015). PVT1: a rising star among oncogenic long noncoding RNAs. Biomed Res Int.

[46] Walz S, Lorenzin F, Morton J (2014). Activation and repression by oncogenic MYC shape tumour-specific gene expression profiles. Nature.

[47] Carramusa L, Contino F, Ferro A (2007). The PVT-1 oncogene is a Myc protein target that is overexpressed in transformed cells. J Cell Physiol.

[48] Tseng YY, Moriarity BS, Gong W (2014). PVT1 dependence in cancer with MYC copy-number increase. Nature.

[49] Zanke BW, Greenwood CM, Rangrej J (2007). Genome-wide association scan identifies a colorectal cancer susceptibility locus on chromosome 8q24. Nat Genet.

[50] Tenesa A, Farrington SM, Prendergast JG (2008). Genome-wide association scan identifies a colorectal cancer susceptibility locus on 11q23 and replicates risk loci at 8q24 and 18q21. Nat Genet.

[51] Tomlinson I, Webb E, Carvajal-Carmona L (2007). A genome-wide association scan of tag SNPs identifies a susceptibility variant for colorectal cancer at 8q24.21. Nat Genet.

[52] Nissan A, Stojadinovic A, Mitrani-Rosenbaum S (2012). Colon cancer associated transcript-1: a novel RNA expressed in malignant and pre-malignant human tissues. Int J Cancer.

[53] Ahmadiyeh N, Pomerantz MM, Grisanzio C (2010). 8q24 prostate, breast, and colon cancer risk loci show tissue-specific long-range interaction with MYC. Proc Natl Acad Sci U S A.

[54] Kim T, Cui R, Jeon YJ (2014). Long-range interaction and correlation between MYC enhancer and oncogenic long noncoding RNA CARLo-5. Proc Natl Acad Sci U S A.

[55] Pomerantz MM, Ahmadiyeh N, Jia L (2009). The 8q24 cancer risk variant rs6983267 shows long-range interaction with MYC in colorectal cancer. Nat Genet.

[56] Sur IK, Hallikas O, Vaharautio A (2012). Mice lacking a Myc enhancer that includes human SNP rs6983267 are resistant to intestinal tumors. Science.

[57] Tuupanen S, Turunen M, Lehtonen R (2009). The common colorectal cancer predisposition SNP rs6983267 at chromosome 8q24 confers potential to enhanced Wnt signaling. Nat Genet.

[58] Xiang JF, Yin QF, Chen T (2014). Human colorectal cancer-specific CCAT1-L lncRNA regulates long-range chromatin interactions at the MYC locus. Cell Res.

[59] Deng L, Yang SB, Xu FF (2015). Long noncoding RNA CCAT1 promotes hepatocellular carcinoma progression by functioning as let-7 sponge. J Exp Clin Cancer Res.

[60] He X, Tan X, Wang X (2014). C-Myc-activated long noncoding RNA CCAT1 promotes colon cancer cell proliferation and invasion. Tumour Biol.

[61] Yang F, Xue X, Bi J (2013). Long noncoding RNA CCAT1, which could be activated by c-Myc, promotes the progression of gastric carcinoma. J Cancer Res Clin Oncol.

[62] Ling H, Spizzo R, Atlasi Y (2013). CCAT2, a novel noncoding RNA mapping to 8q24, underlies metastatic progression and chromosomal instability in colon cancer. Genome Res.

[63] Kim T, Jeon YJ, Cui R (2015). Role of MYC-regulated long noncoding RNAs in cell cycle regulation and tumorigenesis. J Natl Cancer Inst.

[64] Ye Z, Zhou M, Tian B (2015). Expression of lncRNA-CCAT1, E-cadherin and N-cadherin in colorectal cancer and its clinical significance. Int J Clin Exp Med.

[65] Alaiyan B, Ilyayev N, Stojadinovic A (2013). Differential expression of colon cancer associated transcript1 (CCAT1) along the colonic adenoma-carcinoma sequence. BMC Cancer.

[66] Kim T, Cui R, Jeon YJ (2015). MYC-repressed long noncoding RNAs antagonize MYC-induced cell proliferation and cell cycle progression. Oncotarget.

[67] Prensner JR, Chen W, Han S (2014). The long non-coding RNA PCAT-1 promotes prostate cancer cell proliferation through cMyc. Neoplasia.

[68] Yamamura S, Saini S, Majid S (2012). MicroRNA-34a modulates c-Myc transcriptional complexes to suppress malignancy in human prostate cancer cells. PLoS One.

[69] Siemens H, Jackstadt R, Hunten S (2011). miR-34 and SNAIL form a double-negative feedback loop to regulate epithelial-mesenchymal transitions. Cell Cycle.

[70] Benassi B, Flavin R, Marchionni L (2012). MYC is activated by USP2a-mediated modulation of microRNAs in prostate cancer. Cancer Discov.

[71] Poliseno L, Salmena L, Zhang J (2010). A coding-independent function of gene and pseudogene mRNAs regulates tumour biology. Nature.

[72] Salmena L, Poliseno L, Tay Y (2011). A ceRNA hypothesis: the Rosetta Stone of a hidden RNA language?. Cell.

[73] Ge X, Chen Y, Liao X (2013). Overexpression of long noncoding RNA PCAT-1 is a novel biomarker of poor prognosis in patients with colorectal cancer. Med Oncol.

[74] Chung S, Nakagawa H, Uemura M (2011). Association of a novel long non-coding RNA in 8q24 with prostate cancer susceptibility. Cancer Sci.

[75] Hung CL, Wang LY, Yu YL (2014). A long noncoding RNA connects c-Myc to tumor metabolism. Proc Natl Acad Sci U S A.

[76] Chu C, Qu K, Zhong FL (2011). Genomic maps of long noncoding RNA occupancy reveal principles of RNA-chromatin interactions. Mol Cell.

[77] Pickard MR, Williams GT (2015). Molecular and Cellular Mechanisms of Action of Tumour Suppressor GAS5 LncRNA. Genes (Basel).

[78] Hu G, Lou Z, Gupta M (2014). The long non-coding RNA GAS5 cooperates with the eukaryotic translation initiation factor 4E to regulate c-Myc translation. PLoS One.

[79] Mourtada-Maarabouni M, Pickard MR, Hedge VL (2009). GAS5, a non-protein-coding RNA, controls apoptosis and is downregulated in breast cancer. Oncogene.

[80] Pickard MR, Mourtada-Maarabouni M, Williams GT (2013). Long non-coding RNA GAS5 regulates apoptosis in prostate cancer cell lines. Biochim Biophys Acta.

[81] Sun M, Jin FY, Xia R (2014). Decreased expression of long noncoding RNA GAS5 indicates a poor prognosis and promotes cell proliferation in gastric cancer. BMC Cancer.

[82] Tu ZQ, Li RJ, Mei JZ (2014). Down-regulation of long non-coding RNA GAS5 is associated with the prognosis of hepatocellular carcinoma. Int J Clin Exp Pathol.

[83] Li LJ, Zhu JL, Bao WS (2014). Long noncoding RNA GHET1 promotes the development of bladder cancer. Int J Clin Exp Pathol.

[84] Yang F, Xue X, Zheng L (2014). Long non-coding RNA GHET1 promotes gastric carcinoma cell proliferation by increasing c-Myc mRNA stability. FEBS J.

[85] Lemm I, Ross J (2002). Regulation of c-myc mRNA decay by translational pausing in a coding region instability determinant. Mol Cell Biol.

[86] Weidensdorfer D, Stohr N, Baude A (2009). Control of c-myc mRNA stability by IGF2BP1-associated cytoplasmic RNPs. RNA.

[87] Matouk IJ, DeGroot N, Mezan S (2007). The H19 non-coding RNA is essential for human tumor growth. PLoS One.

[88] Matouk IJ, Raveh E, Abu-lail R (2014). Oncofetal H19 RNA promotes tumor metastasis. Biochim Biophys Acta.

[89] Jiang X, Yan Y, Hu M (2015). Increased level of H19 long noncoding RNA promotes invasion, angiogenesis, and stemness of glioblastoma cells. J Neurosurg.

[90] Lottin S, Adriaenssens E, Dupressoir T (2002). Overexpression of an ectopic H19 gene enhances the tumorigenic properties of breast cancer cells. Carcinogenesis.

[91] Luo M, Li Z, Wang W (2013). Long non-coding RNA H19 increases bladder cancer metastasis by associating with EZH2 and inhibiting E-cadherin expression. Cancer Lett.

[92] Ma C, Nong K, Zhu H (2014). H19 promotes pancreatic cancer metastasis by derepressing let-7′s suppression on its target HMGA2-mediated EMT. Tumour Biol.

[93] Zhang EB, Han L, Yin DD (2014). c-Myc-induced, long, noncoding H19 affects cell proliferation and predicts a poor prognosis in patients with gastric cancer. Med Oncol.

[94] Barsyte-Lovejoy D, Lau SK, Boutros PC (2006). The c-Myc oncogene directly induces the H19 noncoding RNA by allele-specific binding to potentiate tumorigenesis. Cancer Res.

[95] Shi Y, Wang Y, Luan W (2014). Long non-coding RNA H19 promotes glioma cell invasion by deriving miR-675. PLoS One.

[96] Kallen AN, Zhou XB, Xu J (2013). The imprinted H19 lncRNA antagonizes let-7 microRNAs. Mol Cell.

[97] Yan L, Zhou J, Gao Y (2015). Regulation of tumor cell migration and invasion by the H19/let-7 axis is antagonized by metformin-induced DNA methylation. Oncogene.

[98] Liao LM, Sun XY, Liu AW (2014). Low expression of long noncoding XLOC_010588 indicates a poor prognosis and promotes proliferation through upregulation of c-Myc in cervical cancer. Gynecol Oncol.

[99] Mestdagh P, Fredlund E, Pattyn F (2010). An integrative genomics screen uncovers ncRNA T-UCR functions in neuroblastoma tumours. Oncogene.

[100] Atmadibrata B, Liu PY, Sokolowski N (2014). The novel long noncoding RNA linc00467 promotes cell survival but is down-regulated by N-Myc. PLoS One.

[101] Tee AE, Ling D, Nelson C (2014). The histone demethylase JMJD1A induces cell migration and invasion by up-regulating the expression of the long noncoding RNA MALAT1. Oncotarget.

[102] Liu PY, Erriquez D, Marshall GM (2014). Effects of a novel long noncoding RNA, lncUSMycN, on N-Myc expression and neuroblastoma progression. J Natl Cancer Inst.

[103] Vadie N, Saayman S, Lenox A (2015). MYCNOS functions as an antisense RNA regulating MYCN. RNA Biol.

[104] Stanton BR, Perkins AS, Tessarollo L (1992). Loss of N-myc function results in embryonic lethality and failure of the epithelial component of the embryo to develop. Genes Dev.

[105] Stanton BR, Parada LF (1992). The N-myc proto-oncogene: developmental expression and in vivo site-directed mutagenesis. Brain Pathol.

